# Monkey pox: Rethinking COVID‐19 to project future strategies against emerging and evolving pathogens

**DOI:** 10.1002/hcs2.20

**Published:** 2022-10-12

**Authors:** Emmanuel Lamptey, Stanley Yaidoo, Ernestina Asiedua, Evans Osei Boakye, Moses tia Banoya, Ephraim Kumi Senkyire

**Affiliations:** ^1^ Institute of Life and Earth Sciences (Including Health and Agriculture), Pan African University University of Ibadan Ibadan Nigeria; ^2^ St. Gregory Catholic Hospital, Central Region Gomoa East District Buduburam Ghana; ^3^ University of Ghana Legon Ghana; ^4^ Department of Social Work University of Ghana Legon Ghana; ^5^ Methodist University Ghana Accra Ghana; ^6^ Ga‐West Municipal Hospital Ghana Health Service Accra Ghana

**Keywords:** COVID‐19, infectious, monkey pox, pandemic

## Abstract

COVID‐19 Monkey pox, days like these need planning

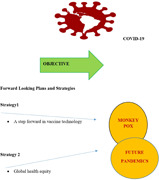

**Abbreviations**
NPIs: non‐pharmacotherapy interventions
R&D: research and development
WHO: World Health Organization

## 1 INTRODUCTION

The COVID‐19 pandemic, which spread quickly across the globe toward the end of 2019 and throughout 2020 impacted almost every aspect of daily life [1]. It generated unprecedented uncertainty in the global economy,and disrupted daily routines. Individuals must navigate quarantines, school closing, job insecurity, significantly impairing the physical, mental, social, financial well‐being of people [2, 3, 4, 5].

The impact of the pandemic was more severe than global financial crisis, which was devastating as well to the health and welfare of the human population [6]. The World Health Organization (WHO) on January 30, 2020 announced that the SARS‐CoV‐2 crisis affected all humans globally with countless fatalities, destabilizing the economy order [7]. Preliminary estimates indicate that the total number of global “excess death” attributable to COVID‐19 in 2020 amount to at least 3 million or higher than official figures tracked by the WHO [8, 9].

At the beginning of the 2022, the pandemic has not relented as there is still a dramatic rise in cases and hospitalizations due to the highly transmissible omicron variant [10]. Increased deaths from other causes due to disruptions to health service delivery and routine immunization, people seeking care, and funding for non‐COVID‐19 services have also been observed [9]. Therefore, pandemic stunned the entire planet with an aftermath of negatively affecting the health sector, distressing the economy, causing social and political crisis, and leaving behind deep scars in the global health system [11, 12, 13]. Despite these consequences, COVID‐19 has shown and exposed many vulnerabilities in the global health system, the implications for future pandemics, and the need for strengthening the capacity of our health systems [8].

The importance of building resilient health systems that will equitably accelerate the shared global goal of responding swiftly and effectively is a potential solution. An ideal future state would be to mount strategies that will strengthen the capacity of these systems and the pandemic has shown how vulnerabilities in health systems can have profound implications for emerging pathogen.

More recently, the monkey pox virus belonging to the genus orthopox family started spreading from Central and Western Africa after first being reported in the Democratic Republic of the Congo in 1970 [14]. Since then, monkey pox has been reported in 11 African countries: Cameroon, the Central African Republic, the Democratic Republic of the Congo, Gabon, Ivory Coast, Liberia, Nigeria, the Republic of Congo, South Sudan, and Sierra Leone [15].

The incidence of monkey pox was reported to be low as 0.64 per 100,000 in 2001 and in 2016 it was 50 per 10,000 cases [16]. Although incidence rates are still low compared to SARS‐CoV‐2, there are case fatalities and the probability spreading to many countries remains high [16]. As of July 23, 2022, the spread of the disease had increased to more than 16,000 cases from 75 countries, causing five deaths [17]. The WHO declared monkey pox a Public Health Emergence of International Concern as it kept spreading around the world rapidly with new modes of transmission and public health measures failed to stem it [17].

Considering COVID‐19 and now the monkey pox, there is a need to plan and learn from contagious diseases to prevent and contain future pandemics. Lessons from the COVID‐19 pandemic are central to this purpose; rethinking preparation and making health systems more capable of handling threats when they emerge. Hence, this paper focuses on the elaboration and recommendations for the directions in which the world must evolve to keep pace with the evolution of emerging contagious diseases.

## 2 A STEP FORWARD IN RESEARCH AND VACCINE DEVELOPMENT TECHNOLOGY

Vaccines are one of the main public health tools and the introduction of the COVID‐19 jabs has significantly changed the epidemiology of the pandemic saving lives and livelihoods. The outbreak demonstrated how the globe had dramatically benefited from their response [7, 18]. In December 2020, the US Food and Drug Administration approved the first COVID‐19 vaccine (Pfizer‐BioNTech) 326 days after SARS‐CoV‐2 emergence [16]. The approval of this high standard, safe and effective vaccine came because of prior research. The vaccines were developed, tested, and approved at a very rapid rate because scientists have studied other coronaviruses for 50 years [19, 20]. Fair knowledge of the SARS‐CoV‐2 spike protein and a decade of mRNA vaccine research enabled the advance of trials of multiple COVID‐19 vaccines at a record speed [19, 20]. The spike proteins genetic codes of were plugged into the already existing technology to make mRNA vaccine candidatures [19, 20]. What scientists learned pivoted them to respond to the COVID‐19 quickly. In short, accelerating the development of vaccines and other biological countermeasures ahead of time provides the next generation of therapeutics to address the global health challenges to the healthcare system. It saves millions of lives, prevents loss from economic damage, and limits the possibility of a new variant emerging. This approach can be replicated for infections such as the monkey pox, other virus families, and future pandemics. With non‐pharmacotherapy interventions (NPIs) such as physical distancing, handwashing, appropriate use of face mask, and homestay for symptomatic patients, this advancement will control transmission as well as increase the level of vaccination coverage in a population [21]. Some degree of transmission control can be achieved with NPIs [21].

## 3 ENSURING GLOBAL HEALTH EQUITY AND PROTECTION FOR VULNERABLE POPULATIONS

An infection anywhere is a potential infection everywhere, because the world is interconnected, and global health problems directly impact others [22]. The novel SARS‐CoV‐2 first reported in Wuhan China had caused millions of deaths within the global population.

Its Delta variant, first identified in India in the late 2020, swept rapidly and became the predominant SARS‐CoV‐2 variant, accounting for more than 99% of COVID‐19 cases and hospitalizations in the United States [23, 24]. Omicron equally spread around the world in a faster mode after 2 months when they were first spotted in South Africa [25]. The WHO stated that cases of monkey pox have been reported from 12 member states not endemic for monkey pox virus on May 13, 2022 [26]. This is evident that there is a weakness in our ability to contain an emerging infectious disease and surveillance efforts have failed as these pathogens sneaked into many countries undetected [22].

Existing disparities in global health systems also contribute to these outcomes [22]. Low‐income countries have higher infection fatality rate than high‐income countries and there is a lower rate of vaccination in these developing countries that is a major problem of global health equity [27, 28]. The burden of COVID‐19 is far higher in developing nations than in the developed due to limited access to adequate healthcare [28]. Subsequently, these countries also have vulnerable groups such as the aged, immunosuppressed, and those with underlying health conditions at higher risk of severe disease and death. There is a critical need to extend global health across reaching this population in these regions [28].

The following stepping stones and future research directions can be adapted. High‐income countries should fund the WHO's essential roles during pandemics [29]. The WHO still faces a funding gap of almost 900 million USD to cover the period of March 2022 [29]. Funding from the International Community enables the WHO to play a global role in tackling and responding to pandemics [29].

In addition to charity from wealth nations, vulnerable people must be prioritized in resources and during outbreak response time [30].

In the COVID‐19 pandemic, vaccines were not reaching many people in developing countries despite all donations and funding [30]. Less than 1% of people in low‐income countries were fully vaccinated and 10% were in lower middle‐income countries that undermine global health equity [30]. The initiation of vaccine and drug patent sharing in developing countries is essential to tackle this problem. In this respect, poorer nations can develop the drugs and vaccines that are needed urgently without depending on richer countries. Various approaches can be deployed to push this agenda. Pharmaceutical and Drug companies licensed their jabs outside their borders to be produced in large quantities in the developing countries or set up manufacturing plants in areas close to the pandemic outbreaks. Supporting R&D and manufacturing of therapeutics in low‐income countries to produce low‐cost vaccines and drugs, especially in Africa, is highly recommended. Coupled with the above‐developing countries must improve early‐stage surveillance of disease outbreaks by close monitoring of incidence, use of digital technologies and prompt laboratory testing and diagnosis of cases [31, 36].

Any infectious disease outbreak must not move faster than the global distribution of vaccines and there should be enough to cover all people globally [33]. Enough doses of vaccines globally can drive down transmission and save lives during a pandemic when they go to the people who need them the most [33].

The initiative to accelerate the development of vaccines will guard against emerging pathogens and diseases that could cause the next devastating endemic pandemic threats [34]. These interventions put forward, with effective communication with the public to disarm misinformation and hesitancy is paramount. Misinformation disparage antivaxxers, conflate large groups of vaccine‐hesitant individuals in developing countries [35]. They are infodemics that make vulnerable people ignore official health guidance during outbreaks, avoid getting vaccinated, or use harmful miracle cures instead of therapeutics [36]. Communicating effectively to establish a rapid response pathway to address public concerns is key. We present these suggestions on strategies for improved future pandemic responses.

## 4 CONCLUSION

Pandemics are constant threats and the emergence of COVID‐19, and currently, monkey pox virus is a clear example. They exposed the cost of glaring global health inequalities. The strategies discussed above indicate that we can learn from the COVID‐19 pandemic to prevent and contain future ones. A comprehensive evaluation of the COVID‐19 outbreak revealed positive progress. We have the tools to bring any pandemic, including monkey pox virus or potential pathogens, under control or accelerate an end to it. To do that, the world must prepare ahead of time and achieve global health equity as this will substantially increase global immunity.

## AUTHOR CONTRIBUTIONS

**Emmanuel Lamptey**: Conceptualization (equal); Data curation (equal); Investigation (equal); Project administration (equal); Validation (equal); Visualization (equal). **Stanley Yaidoo**: Validation (equal); Visualization (equal). **Ernestina Asiedua**: Supervision (equal); Visualization (equal). **Evans Osei Boakye**: Methodology (equal); Resources (equal). **Moses tia Banoya**: Validation (equal); Visualization (equal). **Ephraim Kumi Senkyire**: Validation (equal).

## CONFLICT OF INTEREST

The authors declare no conflict of interest.

## ETHICS STATEMENT

None.

## INFORMED CONSENT

None.

## Data Availability

Data sharing not applicable to this article as no datasets were generated or analyzed during the current study.
